# Cognitive changes and brain connectomes, endocrine status, and risk genotypes in testicular cancer patients–A prospective controlled study

**DOI:** 10.1002/cam4.4165

**Published:** 2021-08-13

**Authors:** Cecilie R. Buskbjerg, Ali Amidi, Mads Agerbaek, Claus H. Gravholt, SM Hadi Hosseini, Robert Zachariae

**Affiliations:** ^1^ Unit for Psychooncology and Health Psychology Department of Psychology and Behavioral Sciences Aarhus University Aarhus Denmark; ^2^ Department of Oncology Aarhus University Hospital Aarhus Denmark; ^3^ Department of Endocrinology Aarhus University Hospital Aarhus Denmark; ^4^ Department of Molecular Medicine Aarhus University Hospital Aarhus Denmark; ^5^ Department of Psychiatry and Behavioral Sciences School of Medicine Stanford University Stanford California USA

**Keywords:** clinical cancer research, genetic variants, survival, urological oncology

## Abstract

**Objective:**

Previous research has indicated cognitive decline (CD) among testicular cancer patients (TCPs), even in the absence of chemotherapy, but little is known about the underlying pathophysiology. The present study assessed changes in cognitive functions and structural brain connectomes in TCPs and explored the associations between cognitive changes and endocrine status and hypothesized risk genotypes.

**Methods:**

Thirty‐eight newly orchiectomized TCPs and 21 healthy controls (HCs) comparable to TCPs in terms of age and years of education underwent neuropsychological testing, structural MRI, and a biological assessment at baseline and 6 months later. Cognitive change was assessed with a neuropsychological test battery and determined using a standardized regression‐based approach, with substantial change defined as z‐scores ≤−1.64 or ≥1.64. MRI scans and graph theory were used to evaluate changes in structural brain connectomes. The associations of cognitive changes with testosterone levels, androgen receptor gene (AR) CAG repeat length, and genotypes (APOE, COMT, and BDNF) were explored.

**Results:**

Compared with HCs, TCPs showed higher rates of substantial decline on processing speed and visuospatial ability and higher rates of substantial improvement on verbal recall and visuospatial learning (*p *< 0.05; OR = 8.15–15.84). Brain network analysis indicated bilateral thalamic changes in node degree in HCs, but not in TCPs (*p *< 0.01). In TCPs, higher baseline testosterone levels predicted decline in verbal memory (*p *< 0.05). No effects were found for AR CAG repeat length, APOE, COMT, or BDNF.

**Conclusions:**

The present study confirms previous findings of domain‐specific CD in TCPs following orchiectomy, but also points to domain‐specific improvements. The results do not indicate changes in brain connectomes or endocrine status to be the main drivers of CD. Further studies evaluating the mechanisms underlying CD in TCPs, including the possible role of the dynamics of the hypothalamic–pituitary–gonadal axis, are warranted.

## Graphical abstract

The present study confirms previous findings of domain‐specific cognitive decline in testicular cancer patients following orchiectomy, but also points to domain‐specific improvements. The results do not indicate changes in brain connectomes or endocrine status to be the main drivers of cognitive changes.

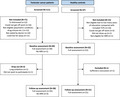

## 1 INTRODUCTION

Over the last decades, increasing evidence of the impact of cancer and cancer treatment on cognition has emerged.^1^ While most of these studies have focused on the specific impact of chemotherapy on cognitive functions in mainly breast cancer populations, only a few studies have investigated cancer‐related cognitive impairment in less common cancers such as testicular cancer and in the absence of systemic therapies. Previous studies have reported (I) cognitive impairment in newly orchiectomized testicular cancer patients (TCPs),^2^, ^3^, ^4^ (II) cognitive decline (CD) in TCPs treated with orchiectomy‐only from baseline to 6 months later compared with healthy controls (HCs),^5^ and (III) similar rates of cognitive impairment in orchiectomy‐only TCPs and TCPs treated with additional chemotherapy, as assessed 2–7 years after orchiectomy.^6^ Together, these findings indicate that TCPs may be at an increased risk of CD unrelated to chemotherapy.

Several studies have documented structural brain alterations in cancer patients. In our previous work, we have demonstrated longitudinal gray matter changes in both TCPs undergoing chemotherapy and surgery‐only TCPs.^5^ We have, furthermore, applied graph theory^7^, ^8^ to demonstrate altered regional connectome properties in newly orchiectomized TCPs receiving no further treatment compared with HCs.^3^ Together, this indicates that altered structural brain organization in TCPs might also be related to other factors than chemotherapy. Graph theory is the mathematical study of graphs that model objects (“nodes”) and their connections (“edges”). In the context of structural brain connectivity, nodes represent brain regions of interest (ROIs) and edges represent structural connections between ROIs.^6^ Graph theory can be used to obtain individual connectome metrics characterizing one or several aspects of global and regional brain connectivity.^6^, ^7^ Such a multivariate approach to brain imaging analysis has distinct advantages compared with more traditional univariate approaches (e.g., voxel‐based analysis) by allowing for the quantification and assessment of the organizational properties of the entire brain connectome. With graph theory, it has been established that brain structural networks follow a specific topology known as small world.^8^ A small‐world network is characterized by a balance between high local clustering of nodes, that is, local segregation, and minimal average path lengths between nodes, that is, global integration, which both enable efficient information processing.^8^

Orchiectomy, the primary treatment for testicular cancer, puts patients at an increased risk of diminished testosterone production,^9^ which could influence the development of CD since evidence suggests that physiological testosterone is important for cognitive functions. Specifically, testosterone appears to be involved in neurophysiological health maintenance, for example, by delaying neuronal apoptosis,^10^ protecting granule cells from oxidative stress,^11^ and reducing beta‐amyloid peptide levels.^12^, ^13^ Furthermore, in vivo studies have demonstrated decreased hippocampal neurogenesis^14^, ^15^ and even demyelination^16^ in castrated rodents. In continuation of these lines of evidence, it has been speculated (e.g.,^17^) that age‐related CD in men^18^ might be influenced by paralleled age‐related decline in testosterone levels.^19^ However, while some studies revealed associations between endogenous testosterone levels and cognitive performance in men (e.g.,^20^, ^21^), others failed to find such associations (e.g.,^22^, ^23^). It has also been indicated that testosterone supplementation may be beneficial for cognitive functions in men; yet, the results of a recent meta‐analysis did not support robust and clinically relevant effects.^24^ In general, the conflicting results from studies investigating the effects of endogenous and supplemented testosterone on cognitive functions in men may partly result from methodological limitations and between‐study variations.^24^, ^25^ For example, when evaluating testosterone levels, it is essential to consider interdependent substances, including sex hormone‐binding globulin (SHBG), and the sensitivity of the androgen receptor (AR), which is primarily determined by a highly polymorphic CAG repeat in exon 1 in the AR gene, which has been found to be inversely correlated with androgen sensitivity.^26^ Notably, it has also been indicated that CAG repeat length may in itself exert an effect on cognitive functions; however, existing findings have been equivocal.^3^, ^26^, ^27^, ^28^, ^29^, ^30^

Given that only a subgroup of cancer survivors develop CD, it is important to elucidate risk factors, including genetic risk.^1^ Previous research has suggested a role for the APOE (e.g.,^5^, ^31^) encoding the glycoprotein apolipoprotein ε, COMT (e.g.,^32^) encoding catechol‐O‐methyltransferase, and BDNF^1^, ^33^ encoding brain‐derived neurotrophic factor. While we have previously reported the APOE ε4 to be a risk factor for CD in TCPs who received chemotherapy,^5^ the vast majority of the available research on genetic risk factors for CD has focused on breast cancer patients.^34^ There is thus a need for studies investigating the role of genotypes in other cancer populations, including TCPs treated with orchiectomy‐only.

Taken together, TCPs may be at risk for CD following orchiectomy, but the underlying pathophysiological mechanisms remain unclear. The aims of the present study were: (I) to compare cognitive changes and brain connectomes in newly orchiectomized TCPs with HCs from a baseline assessment to 6 months later and (II) to explore the associations of cognitive changes with endocrine status and risk genotypes (APOE, COMT, and BDNF).

## 2 MATERIALS AND METHODS

### 2.1 Recruitment and procedures

Newly orchiectomized TCPs were consecutively recruited from February 2018 to September 2019 at the Department of Oncology, Aarhus University Hospital (AUH). Inclusion criteria were confirmed TC diagnosis and orchiectomy with no further treatment (i.e., radiation and/or chemotherapy) received at the time of inclusion. Exclusion criteria included insufficient Danish proficiency, age <18 years, previous cancer or central nervous system disease, known mental disorder, and substance abuse. HCs comparable with TCPs in terms of age and years of education were recruited in the local community through public advertisements. All participants were scheduled for a baseline and 6‐month follow‐up assessment (mean test–retest interval = 190.2 days; SD = 12.3). On average, TCPs were assessed 28 days (SD = 7.7) after unilateral orchiectomy and prior to any further treatment. Assessments at both time points included a questionnaire package, neuropsychological tests, a structural magnetic resonance imaging (MRI) scan, and a biological assessment. At both time points, all assessments were obtained on the same day, and MRI scans were obtained either before or after the neuropsychological assessment. Results of the baseline assessment have been reported elsewhere.^3^

### 2.2 Questionnaires

Questionnaires included sociodemographic factors (e.g., educational status and income) and health behavior outcomes (e.g., alcohol consumption and exercise). Additional patient‐reported outcome measures (PROMs) for commonly reported symptoms included: anxiety and depression,^35^ fatigue,^36^ perceived stress,^37^ and sleep difficulties.^38^ Please refer to Table 1 and Table S1 for further details.

**TABLE 1:** Demographic, clinical, and psychological characteristics of study participants at baseline

	TCP (*N* = 38)	HC (*N* = 21)	*p* value	BCa CI 95%
Demographics				
Age (years), M (SD)	37.7 (12.0)	36.3 (12.1)	0.68	−5.3; 8.1
Education (years), M (SD)	14.9 (2.5)	15.8 (2.7)	0.20	−2.1; 0.3
Premorbid IQ^a^, M (SD)	9.3 (3.2)	11.6 (2.3)	**<*0*.*01*** *	−3.7; −0.7
Occupationally engaged, *N* (%)	30 (78.9)	20 (95.2)	0.14	‐
Income (in 100.000 DKK), M (SD)	4.5 (2.3)	3.9 (2.3)	0.39	−0.7; 1.7
Married/cohabiting, *N* (%)	28 (72.7)	15 (34.9)	0.85	‐
Health behavior				
Exercise (h/week), M (SD)	7.5 (7.75)	4.3 (3.5)	***0*.*05*** *	0.4; 6.1
Body mass index (BMI), M (SD)	26.6 (4.4)	24.9 (4.1)	0.13	−3.6; 0.6
Alcohol (Drinks/week), M (SD)	6.8 (7.06)	4.0 (2.8)	***0*.*03*** *	0.6; 5.3
Smoking (yes), *N* (%)	8 (20.0)	1 (4.5)	0.09	
Clinical variables				
Histology, *N* (%)				
Seminoma, *N* (%)	22 (57.9)	‐	‐	‐
Non‐seminoma, *N* (%)	16 (42.1)	‐	‐	‐
Metastatic involvement (yes)	5 (6.3)	‐	‐	‐
Genotype				
APOE ε4 carrier, *N* (%)	14 (36.8)	3 (14.3)	0.07	‐
COMT Val carrier, *N* (%)	27 (71.1)	14 (66.7)	0.73	‐
BDNF Val/Val carrier, *N* (%)	25 (65.8)	14 (66.7)	0.95	‐
AR CAG repeat length, M (SD)	18.7 (2.6)	19.7 (2.7)	0.69	−1.7; 1.2

Statistically significant group differences (*p* <  0.05; two‐tailed) tested with independent *t*‐test or Chi‐square are marked with * and shown in boldface italic.

Abbreviations: APOE ε, apolipoprotein ε; AR, androgen receptor gene; BCa CI 95%, bias‐corrected and accelerated bootstrapped 95% confidence intervals for difference between means; BDNF, brain‐derived neurotrophic factor; COMT, catechol‐O‐methyltransferase; HC, healthy controls; IQ, intelligence coefficient; N, number of participants; SD, standard deviation; TCP, testicular cancer patients.

^a^ Premorbid IQ was estimated with Wechsler's Adult Intelligence Scale IV^40^ Information subtest scale score.

### 2.3 Neuropsychological assessment

A battery of standardized neuropsychological tests (lasting approximately 1.5 h) was used to assess the cognitive functions in multiple domains. The test battery specifically included the core battery recommended by the International Cancer and Cognition Task Force (ICCTF),^39^ consisting of tests with high sensitivity for measuring cognitive domains that are often impaired in cancer patients while also having good psychometric properties.^39^ In addition to the core battery, we included tests measuring processing speed, attention and working memory, visuospatial ability, and visuospatial learning and memory selected from Wechsler Adult Intelligence Scale‐IV (WAIS‐IV)^40^ and Wechsler Memory Scale III (WMS‐III)^41^: two of the most commonly used neuropsychological test batteries, which are known for good psychometric properties.^42^ In particular, we included several tests measuring visuospatial functions, that is, visuospatial ability and visuospatial learning and memory, as evidence indicates that these functions may be vulnerable to a decline in testosterone levels.^17^ Finally, we included the Wisconsin Card Sorting Test (WCST),^43^ one of the most commonly employed tests for measuring executive functions. The WCST has demonstrated acceptable psychometric properties.^42^, ^44^ Please see Table 2 for further details regarding the test battery and specific cognitive outcomes.

**TABLE 2:** Frequency of decline and improvement in cognitive domains by group

Cognitive domain	Test	Declined, % (*N*)				Improved, % (*N*)			
		TCP (*N* = 38)	HC (*N* = 21)	*p* value	OR (95% CI)	TCP (*N* = 38)	HC (*N* = 21)	*p* value	OR (95% CI)
Processing speed	WAIS‐IV coding (correct)	13.2 (5)	4.8 (1)	0.41	3.00 (0.33–27.84)	2.6 (1)	0.0 (0)	1.00	3.06 (0.14–66.20)
	TMT‐A (s)	28.9 (11)	4.8 (1)	***0*.*03*** *	8.15 (0.97–68.38)	2.6 (1)	4.8 (1)	1.00	0.54 (0.03–9.11)
Attention and working memory	WAIS‐IV digit span (correct)	5.3 (2)	0.0 (0)	0.53	2.95 (0.14–64.26)	5.3 (2)	0.0 (0)	0.53	2.95 (0.14–64.26)
Executive functions	TMT‐B (s)	21.1 (8)	9.5 (2)	0.47	2.53 (0.49–13.23)	15.8 (6)	2.5 (1)	0.40	3.75 (0.42–33.49)
	WCST perseverative errors	18.4 (7)	4.8 (1)	0.24	4.52 (0.52–39.53)	0.0 (0)	0.0 (0)	‐	‐
Verbal fluency	COWA animals (correct)	0.0 (0)	0.0 (0)	‐	‐	13.2 (5)	4.8 (1)	0.41	3.03 (0.33–17.84)
	COWA S (correct)	5.3 (2)	0.0 (0)	0.53	2.95 (0.14–64.26)	0.0 (0)	9.5 (2)	0.12	0.12 (<0.01–2.87)
Verbal learning and memory	HVLT‐R immediate (correct)	10.5 (4)	9.5 (2)	0.64	1.12 (0.19–6.68)	10.4 (4)	0.0 (0)	0.29	5.61 (0.29–109.44)
	HVLT‐R delayed (correct)	7.9 (3)	9.5 (2)	1.00	0.81 (0.13–5.31)	26.3 (10)	0.0 (0)	***0*.*01*** *	15.84 (0.88–285.57)
Visuospatial learning and memory	WMS‐III visual reproduction I (score)	10.5 (4)	0.0 (0)	0.29	5.61 (0.29–109.44)	36.8 (14)	4.8 (1)	***0*.*01*** *	11.67 (1.41–96.60)
	WMS‐III visual reproduction II (score)	26.3 (10)	4.8 (1)	0.08	7.14 (0.85–60.36)	0.0 (0)	0.0 (0)	‐	‐
Visuospatial ability	WAIS‐IV block design (correct)	5.3 (2)	4.8 (1)	1.00	1.11 (0.10–13.03)	2.6 (1)	4.8 (1)	1.00	0.53 (0.03–8.87)
	WAIS‐IV figure weights (correct)	21.1 (8)	0.0 (0)	***0*.*04*** *	11.98 (0.66–218.91)	2.6 (1)	9.5 (2)	0.29	0.25 (0.02–2.94)
	WAIS‐IV matrix (correct)	7.9 (3)	4.8 (1)	1.00	1.71 (0.17–17.60)	2.6 (1)	0.0 (0)	1.00	1.68 (0.07–42.94)
	WAIS‐IV visual puzzles (correct)	13.2 (5)	0.0 (0)	0.15	7.06 (0.37–134.26)	10.5 (4)	4.8 (1)	0.65	2.35 (0.25–22.55)
Overall cognitive decline/improvement		0.0 (0)	0.0 (0)	—	—	0.0 (0)	0.0 (0)	—	—

Statistically significant group differences (*p* < 0.05; two‐tailed) tested with Fischer’s exact test are marked with * and shown in boldface italic.

Abbreviations: %, percentage of impaired participants within group; 95% CI, 95% confidence intervals for difference in counts between groups; COWA, Controlled Oral Word Association phonemic (S) and semantic (animals)^63^; HC, healthy controls; HVLT‐R, Hopkins Verbal Learning Test‐Revised^64^; N, number of participants; OR, odds ratio; TCP, testicular cancer patients; TMT‐A/B, Trail‐Making Test Part A/B^65^; Vis., visual; WAIS‐IV, Wechsler's Adult Intelligence Scale^40^; WCST, Wisconsin Card Sorting Test^43^; WMS‐III, Wechsler's Memory Scale III.^41^

### 2.4 Magnetic resonance imaging

MRI was undertaken for all participants using the same 1.5T Philips Ingenia scanner at both assessments. The acquisition protocol included a T1‐weighted whole‐brain 3D‐TFE sequence, a 32‐directional diffusion‐weighted sequence, and a fluid‐attenuated inversion recovery (FLAIR) sequence. Total scan time was approximately 35 min. The procedure for scan acquisition is specified in the Supplementary Materials.

### 2.5 Biological assessment

At each assessment time point, approximately 10 ml of blood was drawn after an overnight fast and prior to neuropsychological testing between 8.00 and 9.30 AM. Blood samples were processed and serum was prepared according to marker‐specific procedures at the Department of Clinical Biochemistry, AUH.

#### 2.5.1 Sex hormones

Testosterone, estradiol, luteinizing hormone (LH), follicle‐stimulating hormone (FSH), and SHBG were assayed in one batch at the end of the study at the Department of Clinical Biochemistry, AUH. Details regarding these analyses are available in the Supplementary Materials. Free testosterone levels were calculated using Vermeulen's equation.^45^

#### 2.5.2 Hematology

For the assessment of hemoglobin, erythrocytes, and neutrophil counts, approximately 1 ml of blood was assayed immediately at the Department of Clinical Biochemistry, AUH.

#### 2.5.3 Genotyping

Genomic DNA purification, SNP genotyping, and CAG repeat length determination were undertaken at the Department of Molecular Medicine, AUH. Carriers of at least one APOE ε4 allele, at least one COMT VAL allele, and homozygous for the BDNF Val allele, respectively, were classified as risk allele carriers. See Supplementary Materials for further procedural details.

### 2.6 Statistical analysis

IBM Statistical Package for Social Sciences (SPSS) for Windows, version 26.0,^46^ was used for all analyses, with *p* < 0.05 considered to be statistically significant. Between‐group differences across time in commonly reported symptoms, that is, PROMS, were explored with general linear models.

As recommended in the literature,^39^, ^47^, ^48^ longitudinal changes in cognitive performance were analyzed using a standardized regression‐based (SRB) approach,^49^ which enables the adjustment for practice effects, estimated premorbid intelligence, and age. Following this approach, follow‐up cognitive scores in HCs were regressed on their baseline scores, estimated premorbid intelligence, and age. Resulting regression equations were then used to predict all participants' follow‐up scores. Individual z‐scores, indicating direction and magnitude of change of each cognitive outcome, were calculated by subtracting participants' prediction scores from the actual follow‐up scores and dividing with the standard error of estimate of the HC group. The average of all z‐scores was obtained for each participant to get a global composite z‐score (GCS‐z) reflecting the overall cognitive performance across time. Participants with z‐scores ≤−1.64 or ≥1.64, that is, in the extreme 5% at either end of the normal distribution, were classified as demonstrating clinically significant cognitive change. Between‐group differences in clinically significant cognitive changes were compared using Fisher's Exact test,^50^ and odds ratios (ORs) and associated 95% confidence intervals (95% CIs) were calculated. When one or more cells in the contingency table had a value of zero, the Woolf–Haldane Correction^51^ was used to calculate ORs.

Diffusion‐ and T1‐weighted images were used to construct brain connectomes for each participant at each assessment time point. The details of MRI preprocessing, tractography procedure, and brain network construction are given in the Supplementary Materials. Briefly, whole‐brain structural networks were constructed in ExploreDTI,^52^ and a total of 90 ROIs were applied based on the automated anatomical labeling (AAL) atlas.^53^ Networks were then normalized by mean network strength before applying graph theoretical analysis, using the Graph Analysis Toolbox version 1.4.1.^54^

The small‐world organization (small‐worldness, SW) of each network was defined as SW = normalized clustering coefficient (C/C_rand_)/normalized path length (L/L_rand_), where C_rand_ and L_rand_ are the mean clustering and path length of corresponding random networks.^55^ In a small‐world network, the clustering coefficient is significantly higher than that of random networks (C/C_rand_ ratio >1) while the characteristic path length is comparable with random networks (L/L_rand_ ratio close to 1). For a small‐world network, SW should thus be >1.^56^ In addition to the small‐world index, the following global and regional network metrics were calculated: normalized path length, normalized clustering, local and global efficiency, normalized node degree, and betweenness centrality^6^ (see Supplementary Materials for explanation of each network metric). The obtained network metrics were computed across a range of network densities (0.06–0.12) and an AUC measure was calculated. General linear models were used to explore between‐group differences in AUC measures across time. For measures showing between‐group differences, change values (∆) were calculated as the difference between baseline and follow‐up levels.

For each participant, ∆‐values for sex hormones and hematological variables were calculated, and multiple linear regression was used to test for group ×time interactions using baseline values, group, and the interaction variable as predictors.

Linear regression models were also used to explore the following predictors of clinically significant cognitive changes: ∆‐anxiety, ∆‐depression, ∆‐fatigue, ∆‐perceived stress, ∆‐sleep difficulties, ∆‐network values, baseline total and free testosterone levels, ∆‐total testosterone, ∆‐free testosterone, ∆‐estradiol, and CAG repeat length. In case of statistical significance, subsequent interaction tests were performed. Multiple regression models were used to explore the baseline total testosterone levels/∆‐testosterone as predictors of cognitive performance when adjusting for baseline SHBG levels/∆‐SHBG and CAG repeat length. Finally, for TCPS, the possible effect of risk genotypes was explored with linear regression models.

## 3 RESULTS

**FIGURE 1:**
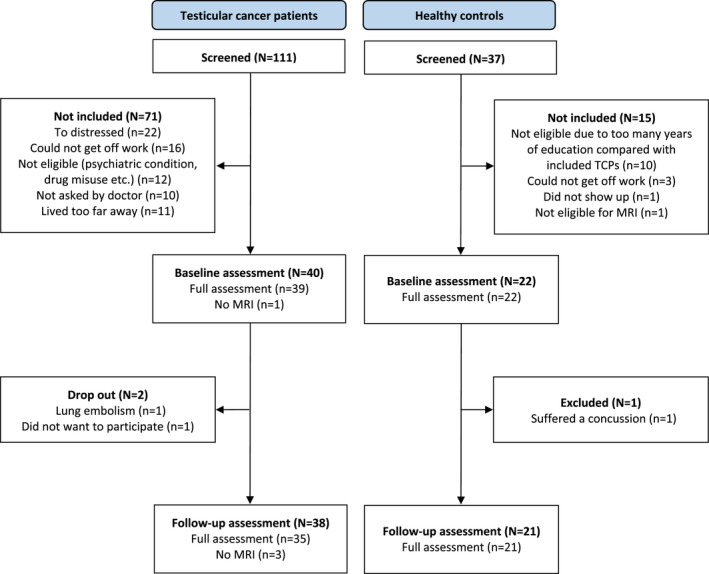
Study flow diagram. Abbreviations: MRI, magnetic resonance imaging; n, number; TCPs, testicular cancer patients

Of 99 eligible TCPs, 40 agreed to participate (approximately 40%). There were no statistically significant clinical or demographical differences between participating and non‐participating patients. Two patients declined to participate in the follow‐up assessment, and 38 patients were thus included in the final analyses (Figure 1). Thirty‐three TCPs presented with stage I disease, and five presented with metastatic disease. At the follow‐up assessment, four metastatic patients had received three rounds of the combined cytostatic regimens of bleomycin, etopside, and cisplatin, and one had received four rounds of etopside and cisplatin. Twenty‐two men were enrolled in the HC group. One HC suffered a concussion prior to the follow‐up assessment and was excluded. All participants underwent full assessment, except one TCP who did not have the MRI scans due to claustrophobia, and two TCPs who did not have the follow‐up MRI scan due to scheduling issues. With the exception of these three MRI scans and two further MRI scans that were excluded from the brain network analysis (see “Brain network analysis below”), there were no missing data in the present study. There were no significant between‐group differences for any demographic variables. However, TCPs performed poorer than HCs on the test of premorbid intelligence and consumed more alcohol (Table 1). Furthermore, there were no between‐group differences in changes across time in commonly reported symptoms (PROMS) (Table S1).

### 3.1 Cognitive changes

Compared with HCs, the percentage of TCPs demonstrating CD was statistically significantly higher for the TMT‐A and WAIS‐IV Figure Weights (OR = 8.15 and 11.98, respectively) (Table 2). For the remaining 13 tests, a higher percentage of TCPs than HCs demonstrated CD for 11 tests. The differences, however, did not reach statistical significance (*p* = 0.08–1.00; OR = 1.11–7.06). Concerning improvement, statistically significantly more TCPs than HCs improved on the HVLT Delayed and WMS‐III Visual reproduction I (OR = 15.84 and 11.67, respectively). For the remaining test, a higher percentage of TCPs than HCs demonstrated improvement on nine tests. Again, the differences did not reach statistical significance (*p* = 0.29–1.00; OR = 2.35–5.61) (Table 2). When five patients with metastatic disease were excluded, the results did not change, with the exception that the difference in percentage of TCPs demonstrating CD no longer reached statistical significance for WAIS‐IV Figure Weights (15.2% vs. 0%; OR = 8.30).

### 3.2 Brain network analysis

One TCP and one HC were excluded from the brain network analysis due to corrupted diffusion‐weighted sequences at the follow‐up assessment. Small‐world organization (SW > 1) was evident in all participants. Statistically significant group × time interaction effects were found for node degree in the right thalamus and left thalamus, respectively (*p* < 0.01, corrected for false discovery rate [FDR]). Results indicated a large decline in left thalamus for HCs (∆ = −3.59; SD = 3.49) compared with TCPs (∆ = −0.20; SD = 4.11), and a large increase in right thalamus for HCs (∆ = 4.53; SD = 3.83) compared with TCPs (∆ = 0.49; SD = 3.08). No significant between‐group differences across time were observed for the tractography and global brain network measures (Table 3).

**TABLE 3:** Between‐group differences across time in DTI tractography and brain network measures

	Baseline (T1) M (SD)		Follow‐up (T2) M (SD)		*p* value
	TCP (*N* = 33)	HC (*N* = 20)	TCP (*N* = 33)	HC (*N* = 20)	
DTI tractography^a^, M (SD)					
Tract length (mm)	100.01 (6.45)	99.69 (4.58)	99.30 (6.53)	100.58 (4.23)	0.12
Number of tracts	16.85 (2.45)	16.61 (2.34)	16.82 (2.39)	16.64 (2.57)	0.94
Fractional anisotropy	0.39 (0.01)	0.39 (0.01)	0.39 (0.01)	0.39 (0.01)	0.27
Network analysis (AUC)^b^, M (SD)					
Normalized clustering	12.21 (1.44)	12.12 (1.44)	12.02 (1.06)	12.31 (1.23)	0.44
Normalized path length	6.58 (0.13)	6.54 (0.09)	6.56 (0.12)	6.55 (0.13)	0.47
Small‐worldness index	11.12 (1.18)	11.12 (1.25)	10.98 (0.87)	11.32 (1.01)	0.48
Global efficiency	2.31 (0.12)	2.29 (0.16)	2.29 (0.17)	2.34 (0.12)	0.29
Local efficiency	2.55 (0.25)	2.51 (0.22)	2.51 (0.30)	2.61 (0.27)	0.15
Node degree (AUC)^b^, M (SD)					
Left thalamus	9.01 (2.67)	12.59 (3.05)	9.21 (3.01)	9.00 (3.07)	**<*0*.*01*** *
Right thalamus	12.42 (3.01)	8.57 (2.23)	12.92 (1.9)	13.19 (2.58)	**<*0*.*01*** *

Statistically significant group differences (*p* < 0.05; two‐tailed) tested with repeated‐measures analysis of variance are marked with * and shown in boldface italic. For node degree, *p* values were corrected for false discovery rate (FDR).

Abbreviations: AUC, area under the curve; HC, healthy controls; M, mean; N, number of participants; SD, standard deviation; T1, values at baseline; T2, values at follow‐up; TCP, testicular cancer patients.

^a^ Average between two regions of interest (ROIs), which each constitutes a node in the brain network.

^b^ Area under the curve (AUC) across a range of densities (0.06–0.12). Values were multiplied by 100.

### 3.3 Endocrine status and hematology

Compared with HCs, TCPs showed increase in SHBG levels and reduction in neutrophil counts (Table 4). Group x time effects were found for neutrophil counts (β = 0.57; *p* < 0.01). To account for the increase in SHBG, the major testosterone protein carrier in serum, we calculated free testosterone levels and observed lower levels at follow‐up in TCPs (M = 0.29 nmol/L; SD = 0.08) compared with HCs (M = 0.38 nmol/L; SD = 0.10) (*p* < 0.01). No between‐group differences were found for mean CAG repeat length (Table 1), and CAG repeat length was not significantly associated with sex hormone change values (data not shown).

**TABLE 4:** Endocrinology and hematology baseline and change scores by group

Analysis	Baseline (T1) M (SD)		*p* value	BCa 95% CI	Change score (T2‐T1)		*p* value	BCa 95% CI
	TCP (*N* = 38)	HC (*N* = 21)			TCP (*N* = 38)	HC (*N* = 21)		
Testosterone (nmol/L), M (SD)	15.87 (5.40)	19.36 (5.90)	***0*.*03*** *	−6.41, −0.74	1.67 (4.77)	0.41 (5.54)	0.39	−4.38; 1.24
Free testosterone (nmol/L), M (SD)	0.30 (0.08)	0.38 (0.09)	***0*.*04*** *	−0.13, −0.02	−0.01 (0.06)	−0.01 (0.09)	0.74	−0.04; 0.03
Estradiol (pmol/L), M (SD)	66.62 (52.14)	69.89 (30.67)	0.76	−23.13, 18.47	30.87 (67.47)	22.29 (48.10)	0.58	−20.94; 33.77
SHBG (nmol/L), M (SD)	38.18 (16.34)	41.43 (26.44)	0.47	−12.17, 4.49	9.11 (22.83)	0.45 (7.34)	***0*.*04*** *	2.08; 17.95
LH (IU/L), M (SD)	9.94 (7.90)	6.16 (2.72)	***0*.*01*** *	1.38, 6.67	−0.44 (8.62)	0.60 (2.19)	0.49	−4.93; 1.76
FSH (IU/L), M (SD)	13.13 (10.55)	6.60 (7.43)	**<*0*.*01*** *	2.18, 10.63	3.18 (9.24)	0.58 (1.23)	0.10	−0.45; 5.52
Hemoglobin (mmol/L), M (SD)	9.10 (0.52)	9.38 (0.51)	**<*0*.*05*** *	−0.57, −0.02	0.08 (0.67)	0.10 (0.54)	0.92	−0.33; 0.31
Erythrocytes (× 10<sup>9</sup>/l)	0.43 (0.02)	0.45 (0.02)	**<*0*.*01*** *	−0.03, −0.01	<0.01 (0.03)	<0.01 (0.02)	0.87	−0.02; 0.01
Neutrophils (× 10<sup>9</sup>/l)	3.67 (1.96)	2.55 (1.07)	**<*0*.*01*** *	0.41, 1.91	−0.81 (1.54)	0.06 (0.53)	***0*.*02*** *	−1.43; −0.35

Note

Change values were calculated as baseline levels subtracted from follow‐up levels.

Statistically significant group differences (*p* < 0.05; two‐tailed) tested with independent *t*‐tests are marked with * and shown in boldface italic.

Abbreviations: BCa CI 95%, bias‐corrected and accelerated bootstrapped 95% confidence intervals for differences in group mean change scores; FSH, follicle‐stimulating hormone; HC, healthy controls; LH, luteinizing hormone; M, mean; N, number of participants; SD, standard deviation; SHBG, sex hormone‐binding globulin; T1, values at baseline; T2, values at follow‐up; TCP, testicular cancer patients.

### 3.4 Predictors of cognitive changes

In HCs, higher ∆‐node degree in left thalamus predicted higher HVLT‐R Delayed z‐scores (β = 16.37) and higher ∆‐node degree in right thalamus predicted higher z‐scores for HVLT‐R Delayed (β = 16.38), TMT‐A (β = 13.92), and WAIS‐IV Figure Weights (β = −15.80) (all *p* < 0.01). Thalamic ∆‐node degree did not predict the cognitive changes in TCPs. Formal group × predictor tests reached significance for left thalamus as a predictor of HVLT‐R Delayed (*p* = 0.03), but not for right thalamus as a predictor of HVLT‐R Delayed (*p* = 0.05), TMT‐A (*p* = 0.31), or WAIS‐IV Figure Weights (*p* = 0.07). In TCPs, lower total and free testosterone levels at baseline predicted higher HLVT‐R Delayed z‐scores (β = −0.10; *p* = 0.03 and β = −7.18; *p* = 0.02). Formal group × predictor tests did not reach statistical significance. None of the remaining investigated predictors of cognitive changes reached statistical significance in neither TCPs, nor HCs. When adjusting for baseline or change in SHBG levels and CAG repeat length, neither baseline levels, nor changes in total testosterone predicted cognitive changes in TCPs (*p* = 0.18–0.99). In TCPs, no effects of APOE, COMT, or BDNF were found for clinically significant cognitive changes (*p* = 0.17–0.75).

## 4 DISCUSSION

We have previously reported baseline data from the present sample indicating that a high proportion of newly orchiectomized TCPs showed signs of cognitive impairment when compared with HCs.^3^ The present study further adds to this by demonstrating that–when compared with HCs–a number of TCPs may also show further CD during the first 6–7 months following orchiectomy. Overall, TCPs evidenced greater decline on two tests related to processing speed and visuospatial ability. In addition, a high percentage of TCPs also demonstrated improvement on two tests related to verbal recall and visuospatial learning. Group‐level analysis at baseline revealed significantly poorer performance in TCPs compared with HCs on these two tests.^3^ Accordingly, TCPs had more room for improvement on these two tests compared with HCs. On the other hand, there is evidence showing that while some cancer patients exhibit persistent cognitive impairment, others may improve in cognitive performance in the months following treatment,^57^, ^58^ suggesting that the effects of the disease and treatment may be transient in these patients. Overall, these results indicate diversity in cognitive development during the first 6–7 months following orchiectomy in the present TCP sample, with some showing CD and others showing improvement. Notably, these results did not change when five TCPs who had received chemotherapy were excluded from the analyses.

A basic property of brain networks is node degree, indicating the number of incoming connections (edges) each node has with the rest of the network and, thus, the centrality of the node to the overall network.^7^ Our results revealed between‐group differences in changes across time in node degree for right thalamus and left thalamus, respectively. A previous study reported gray matter reductions in thalamus in breast cancer patients shortly after surgery, indicating that surgical procedures may specifically affect this brain region.^59^ However, surprisingly, our results indicated that HCs evidenced increase in the left thalamus and decrease in the right thalamus, whereas changes in TCPs were negligible. While we do not have a clear explanation for these findings, the thalamus is believed to act as a relay for information between subcortical and cerebral areas, and thalamic lesions have been associated with impaired learning and memory abilities.^42^ Consistently, in HCs, increased bilateral thalamic node degree predicted improved verbal memory. Furthermore, increased right thalamic node degree predicted improved processing speed and visuospatial ability, the latter being consistent with neuroimaging studies indicating that right thalamic regions are important for visuospatial functions.^42^ While these results are interesting from a more general neurological perspective, they do not support that altered brain connectomes underlie CD in TCPs.

In contrast to our hypothesis that orchiectomy‐related testosterone level decline would contribute to CD in TCPs, changes in testosterone levels failed to predict cognitive changes in the present sample. Baseline testosterone levels were significantly lower in TCPs than in HCs, and we, therefore, also explored baseline testosterone levels as possible predictors of cognitive change and found that higher baseline testosterone levels predicted greater decline in verbal memory in TCPs. While we do not have a clear explanation for this unexpected inverse association, previous research has indicated a nonlinear association between testosterone levels and verbal memory in older men.^60^, ^61^ Such a possible nonlinear task‐specific relationship, however, remains controversial. In line with the results of studies with healthy adults^26^, ^28^ and men with Klinefelter syndrome,^27^ AR CAG repeat length did not predict the cognitive change in TCPs in the present study. In contrast, we have previously reported an association between high CAG repeat length and better verbal memory performance at the baseline assessment of the present TCP sample.^3^ This finding, however, was in contrast with previous studies with prostate cancer patients^30^ and healthy older men^29^ reporting associations between high CAG repeat length and poorer cognitive functions. Accordingly, given the limited and conflicting evidence, it remains unclear how CAG repeat length might exert an effect on cognitive functions in different populations.

While previous research suggests that APOE, COMT, and BDNF polymorphisms may increase the risk for CD in cancer patients,^1^, ^34^ we observed no significant associations between these genes and CD in TCPs. One possible explanation may be that previous research has mainly focused on patients undergoing chemotherapy, which was only the case for a subgroup of patients in the present sample.

### 4.1 Study strengths and limitations

Some limitations should be taken into account when interpreting the present results. First, we report uncorrected multiple testing of associations between cognitive changes and possible predictors, that is, commonly reported symptoms (PROMS), endocrine factors, hematological variables, and risk genotypes. This may be justified by the dependent and exploratory nature of our analyses but should be taken into account when interpreting our results. Second, the sample size of the HC group was smaller than the TCP group, which may have limited our ability to detect associations within this group. Finally, the study participation rate was relatively low, with many patients feeling too distressed or unable or reluctant to take a day off to participate in the study. Our study, however, also has several strengths, including a prospective design, the inclusion of demographically comparable HCs, a low attrition rate (5%), state‐of‐the‐art analysis of cognitive changes, MRI data, and endocrine status.

### 4.2 Clinical implications

Despite the limitations, our findings support that cancer‐related CD–often referred to by patients and clinicians as “chemo‐brain”–is a multifactorial phenomenon not only caused by chemotherapy. While most of the existing research on CD in TCPs has focused on the adverse effects of chemotherapy, given that at least 50% of all TCPs do not receive further treatment than orchiectomy,^62^ the occurrence of CD in this group calls for attention. Importantly, our findings also seem to indicate that some TCPs improve in cognitive functions in the months following orchiectomy, revealing heterogeneity in the cognitive development following surgery for testicular cancer. Increased awareness of the existence of CD in TCPs treated with orchiectomy‐only and of heterogeneity in its development may better equip health care professionals for identifying and guiding patients suffering from these symptoms.

### 4.3 Conclusions

In conclusion, our study provides further evidence for domain‐specific CD in TCPs in the months following orchiectomy, but also points to domain‐specific cognitive improvements in some patients. Our results do not support that altered brain connectomes, APOE, COMT, or BDNF are important for cognitive changes. Moreover, while the present results do not support a clinically relevant impact of testosterone levels or other endocrine factors, future studies could further investigate the possible role of orchiectomy‐related disruption of the dynamics of the hypothalamic–pituitary–gonadal axis. Indeed, if we are to develop efficient preventive and treatment strategies for CD in cancer patients, including TCPs, it is important to expand our understanding of the possible risk factors and underlying pathophysiological mechanisms.

## DATA AVALIABILITY STATEMENT

The data that support the findings of this study are available upon request from the corresponding author. The data are not publicly available due to privacy or ethical restrictions.

## CONFLICT OF INTERESTS

The authors declare no competing financial interests.

## ETHICAL APPROVAL STATEMENT

The study was approved by the Regional Scientific Ethical Committee for Central Denmark Region (No. 1–10–72–406–17). Data were collected and stored in accordance with the Danish Data Protection Agency guidelines, and written informed consent was obtained from all participants upon enrollment. The study was performed in accordance with the Declaration of Helsinki.

## CLINICAL TRIAL REGISTRATION NUMBER

The study was pre‐registered on clinicaltrials.org (#NCT03452436).

## Supporting information

Supplementary Material
