# Supplementary material for: Cognitive changes and brain connectomes, endocrine status, and risk genotypes in testicular cancer patients–A prospective controlled study
Source: Cancer Med. 2021 Aug 13;10(18):6249–60. doi: 10.1002/cam4.4165 (PMC8446403; doi:10.1002/cam4.4165)
Supplement: Supplementary file 1 — Supplementary Material [file CAM4-10-6249-s001.docx]

**SUPPLEMENTARY MATERIAL**

Buskbjerg CR, Amidi A, Agerbæk M, Gravholt CH, Hosseini SMH, Zachariae R. Cognitive Changes and Brain Connectomes, Endocrine Status, and Risk Genotypes in Testicular Cancer Patients – A Prospective, Controlled Study.

Corresponding author:

Cecilie R Buskbjerg

Bartholins Allé 11, building 1351, DK-8000 Aarhus C, Denmark

E-mail: cdrc@psy.au.dk; Phone: +45 29642526

**TABLE OF CONTENTS**

**Supplementary methods………………………………………….…………………………………………….………….…….……**2

Cognitive changes…………………………………………………….……………………………………….……….………….…….……2

Magnetic resonance imaging.…………………………………………………………………………………….……………..………2

*Scan acquisitions and preprocessing….…………..……………….……………………………………….…………..………2*

*Processing and tractography……..……….……………..…………………………………………………………………………3*

*Brain network construction………………………………..…………………………….…………………………………………..3*

*Brain network analysis……………………….………………….………………………………………..……………………………3*

*Description of brain network measures…………………………………………………………………………………………4*

Sex hormone analysis…………….…………………………………………………………………..……………………………………..4

Genotyping………………………..….…………………………………………………………………..………………………………………4

Androgen receptor gene CAG repeat length ………………………..….……………………….........….……………………5

**Supplementary Table S1……………………………………………………………………………………………………..…………6 References………………………………………………………………………….……………………..…………….…………………..7**

**SUPPLEMENTARY METHODS**

**Magnetic resonance imaging**

*Scan acquisitions and preprocessing*

Magnetic Resonance Imaging (MRI) was undertaken using a 1.5T scanner (Philips Ingenia) with a 32-channel phased-array head coil. Total scan time was approximately 35 minutes. The acquisition protocol included a T1-weighted whole brain 3D-TFE sequence with 200 continuous sagittal slices; slice thickness = 1.0 mm, FOV = 256 × 256 mm^2^, TR = 7.5 ms, TE = 3.4 ms, flip angle = 8°, matrix = 256 × 256, voxel size = 1.0 mm isotropic, and 2 excitations. In addition, a 32-directional diffusion-weighted sequence was performed using a single-shot spin-echo echoplanar imaging sequence with 60 contiguous slices, FOV = 240 × 240 mm^2^, matrix size = 128 × 128 mm^2^, TR = 8000 ms, TE = 81 ms, slice thickness = 3 mm, 1.9 × 1.9 × 3 mm^3^ voxel size. Images were acquired in the axial plane with diffusion gradients applied in 32 non-collinear directions with a *b-*value of 1000 s/mm^2^ and one non-diffusion-weighted (*b* = 0) image. Finally, a fluid-attenuated inversion recovery (FLAIR) sequence was performed to search for brain pathology with 22 transverse slices; slice thickness = 5.0 mm; FOV = 352 × 352 mm^2^; TE = 140 ms; TR = 11.000 ms. Minimal movement of participants’ heads during the scan was ensured with customized plastic foam molds.

*Processing and tractography*

Diffusion-weighted images (“diffusion tensor imaging”; DTI), were each visually checked and diffusion volumes with visible artifacts such as venetian blinds were omitted from the subsequent estimation of diffusion parameters. ExploreDTI^1^ was used to perform DTI preprocessing, and subject motion and eddy current induced geometric distortions was corrected by reorienting the B-matrix as described by Leemans & Jones (2009)^2^. A correction procedure based on total variation^3^ were used to correct for Gibbs ringing artifacts. EPI susceptibility distortions were corrected by registering the participant’s diffusion data to the participant’s own undistorted T1-weighted structural image as described by Irfanoglu and colleagues (2012)^4^. An iterative nonlinear tensor estimation process was used to generate fractional anisotropy (FA) maps and whole brain tractography was performed in participant native space for each participant using a deterministic streamline approach^5^ (tracking threshold: 0.2; angle threshold*:* 45 degrees; fiber length threshold: 1-500 mm).

*Brain network construction*

ExploreDTI was used to construct individual brain networks. First, the automated anatomical labeling (AAL) atlas^6^ was used to parcellate the whole-brain tractography of the previous step. Following this approach, the Montreal Neurological Institute brain template is divided into 90 cortical and subcortical regions of interest (ROI), which each constitutes a node in the brain network. ROIs were considered connected when a reconstructed fiber tract started in one region and ended in another one. The ROIs from the AAL atlas, defined in MNI space, were transformed to native space using the T1 weighted image. For each participant, two weighted 90 × 90 connectivity matrices were obtained, of which one matrix was weighted by the number of reconstructed tracts, while the other was weighted by the mean FA value. As in our previous study^7^, a threshold of three tracts was applied to the first matrix in order to minimize false-positive edges^8,9^. Finally, a single connectivity matrix with a product weighting of tract number and mean FA was obtain by multiplying the remaining valid edges with the mean FA values.

*Brain Network Analysis*

Graph analysis was undertaken using The Graph Analysis Toolbox version 1.4.1.^10^. First, the weighted connectivity matrices were normalized by the mean network strength and then thresholded over a range of network densities (0.06 to 0.12 in steps of 0.01). The lower network density limit was set to ascertain none of the individual networks are fragmented and the computed network metrics are not biased^11,12^. The upper density was exerted by the maximum density across individual networks. Global and regional network measures were then quantified for the thresholded networks, including: characteristic path length (L), network clustering coefficient (C), local and global efficiency, normalized node degree, and betweenness centrality. Small-world index (SW) of each network was computed as SW = (C/C_rand_) / (L/L_rand_), where C_rand_  and L_rand_ are the mean clustering and path length of 20 null networks with the same number of nodes, degrees and degree distributions as the actual networks^13^. For each network measure, an area under the curve (AUC) summary measure, which is less sensitive to thresholding and reduces the number of comparisons, was computed. General linear models were used to explore between-group differences in AUC measures across time.

*Description of brain network measures*

Graph theoretical analyses have shown that brain structural networks exhibit small-world characteristics: a topology characterized by a balance between a high clustering of nodes and short average path lengths between nodes^14^. Accordingly, two central properties of small-worldness are the clustering coefficient and the characteristic path length^15,16^. The *clustering coefficient* of a node is defined as the fraction of immediate neighboring nodes that are interconnected, and the *characteristic path* length is the average shortest path length (i.e., sequences of distinct nodes and edges) between all pairs of nodes within the network^15,16^. *Local efficiency* indicates how effectively information is integrated between the immediate neighbors of a node, and is defined as the inverse of the average shortest path connecting all neighbors of a node^17,18^. *Global efficiency* indicates how effectively information is integrated across the entirety of the network and is inversely related to the characteristic path length^18^. The two most common regional outcomes include node degree and betweenness centrality. *Node degree* indicates the amount of incoming connections (edges) each node has with the rest of the network, and is defined as the number of edges connected to a given node^15^. *Betweenness centrality* defines the fraction of all the shortest paths in the network that pass through a given node, and indicates the importance of a node to the overall network integrity^15^.

**Sex hormone analysis**

Total testosterone and estradiol were measured by liquid chromatography tandem mass spectrometry (LC-MS/MS): working range = 0.12-300 nmol/L (testosterone) and 15-15000 pmol/L (estradiol); coefficient of variation of 7.0-10.0 % (testosterone) and 7.5 % (estradiol). Luteinizing hormone (LH), follicle stimulating hormone (FSH), and sex hormone binding globulin (SHBG) were analyzed by electrochemiluminescence (ECLIA) employing the Cobas 8000 instrument (Cobas, Roche Diagnostics Limited, Rotkreuz, Switzerland). Working ranges were 0.3-200 IU/L for LH and FSH, and 0.9-2000 nmol/L for SHBG, and coefficient of variation was 1.6-2.2% for LH, 3.6-4.5% for FSH, and 1.8-4.0% for SHBG.

**Genotyping**

Genomic DNA was purified from 400 µl peripheral blood using DSP DNA Midi Kit (ID: 937255) and QIAsymphony SP (Qiagen, Hilden, Germany) according to the manufacturer’s instructions. The quality of DNA samples were quantified using Qubit fluorometric quantification (dsDNA BR assay) (ThermoFisher, Waltham, MA, USA). Finally, the DNA was diluted with water to 5 ng/µl, and stored at −20°C. Genotyping of SNPs in *APOE* (rs7412C/T (Arg/Cys) and rs429358T/C (Cys/Arg), the two SNPs responsible for the three *APOE* isoforms), *COMT* (rs4680G/A (Val/Met)) and *BDNF* (rs6265C/T (Val/Met)) were carried out with 10 ng of genomic DNA and using the following TaqMan SNP Genotyping assays: C_904973_10, (rs7412), C_3084793_20 (rs429358), C_25746809_50 (rs4680), and C_11592758_10 (rs6265) (ThermoFisher, Waltham, MA, USA). The genotyping assays were performed in duplicates on a ViiA 7 Real time PCR instrument (Applied Biosystems, Thermofisher, Waltham, MA, USA) according to the manufacture´s recommendations. Data analyses and genotyping from allelic discrimination plots were performed using in ViiA TM 7 software (ThermoFisher, Waltham, MA, USA). Carriers of at least one *APOE* ε4 allele (rs7412: C allele (Arg) and rs429358: C allele (Arg)) were classified as risk carriers, as were carriers of at least one *COMT* G allele (Val/Val or Val/Met), and homozygous for the *BDNF* C allele (Val/Val).

**Androgen receptor gene CAG repeat length**

Androgen receptor gene (*AR*) CAG repeat length was determined by PCR amplification on a 96-Well Thermal Cycler (Applied Biosystems). Primers AR-F 5’-FAM-AAGTGATCCAGAACCCGGG-3’ and AR-R 5’- CTCATCCAGGACCAGGTAGC-3’ were chosen to amplify a region containing the repeat polymorphism. The reaction mixture contained 50 ng DNA, 4 pmol of each primer, 1.5 µL 1.25 mM dNTP, 1.5 µL 10x Expand High Fidelity Buffer with 15 mM MgCl_2_ (Roche) and 0.35 U of Expand High Fidelity Enzyme mix (Roche). The PCR conditions were 95^o^C for 4 min, followed by 5 cycles of 95^o^C for 15 s, 58^o^C for 30 s, and 72^o^C for 45 s, followed by 35 cycles of 95^o^C for 15 s, 52^o^C for 30 s, and 72^o^C for 45 s, and finally 74^o^C for 10 min. PCR fragment length was determined by capillary electrophoresis on a 3500 Genetic analyzer (Applied Biosystems) using a Genescan 400HD ROX ladder (Applied Biosystems). Finally, *AR* CAG repeat length was calculated based on the predicted PCR fragment length of 242 bp from the human reference genome (hg19), containing 22 CAG repeats.

**Supplementary Table S1.** Between-group differences across time in psychological symptoms

|  | Baseline (T1) M (SD) | | Follow-up (T2) M (SD) | |  |
| --- | --- | --- | --- | --- | --- |
|  | TCP (N=38) | HC (N=21) | TCP (N=38) | HC (N=21) | *p*-value |
| Anxiety (HADS) | 5.47 (3.22) | 5.19 (3.17) | 5.08 (2.72) | 4.95 (2.97) | 0.79 |
| Depression (HADS) | 2.00 (2.63) | 2.00 (1.61) | 2.18 (2.60) | 2.62 (3.32) | 0.73 |
| Fatigue (FACIT) | 43.24 (6.84) | 46.24 (5.00) | 42.82 (9.17) | 44.38 (7.62) | 0.20 |
| Perceived stress (PSS-10) | 12.37 (6.84) | 8.95 (5.65) | 9.95 (5.59) | 8.24 (4.50) | 0.80 |
| Sleep quality (PSQI) | 7.11 (3.04) | 5.67 (2.22) | 5.61 (3.56) | 4.67 (2.31) | 0.10 |

*Abbreviations*: HC = Healthy controls; N = number of participants; SD = standard deviation; TCP = testicular cancer patients.

HADS = The Hospital Anxiety and Depression Scale^19^; higher scores = higher levels of anxiety/depression.

FACIT = The FACIT Fatigue Scale^20^; higher scores = lower levels of fatigue.

PSS-10 = Perceived Stress Scale, 10 items^21^; higher scores = higher levels of distress.

PSQI = Pittsburgh Sleep Quality Index^22^; higher scores = higher levels of sleep disturbance.

^*^Statistical significance = *p*<0.05 (two-tailed) tested with repeated measures ANOVA.

**REFERENCES**

1. Leemans A, Jeurissen B, Sijbers J, Jones D. ExploreDTI: A graphical toolbox for processing, analyzing, and visualizing diffusion MR data. Proceedings of the 17th Scientific Meeting, International Society for Magnetic Resonance in Medicine 2009;3537.

2. Leemans A, Jones DK. The B-matrix must be rotated when correcting for subject motion in DTI data. Magn Reson Med 2009;61:1336-1349.

3. Perrone D, Aelterman J, Pižurica A, Jeurissen B, Philips W, Leemans A. The effect of Gibbs ringing artifacts on measures derived from diffusion MRI. NeuroImage 2015;120:441-455.

4. Irfanoglu MO WL, Sarlls J, Marenco S, Pierpaoli C. Effects of image distortions originating from susceptibility variations and concomitant fields on diffusion MRI tractography results. Neuroimage 2012;61:375-388.

5. Basser PJ PS, Pierpaoli C, Duda J, Aldroubi A. In vivo fiber tractography using DT-MRI data. Magn Reson Med 2000;44:625-632.

6. Tzourio-Mazoyer N, Landeau B, Papathanassiou D, et al. Automated Anatomical Labeling of Activations in SPM Using a Macroscopic Anatomical Parcellation of the MNI MRI Single-Subject Brain. NeuroImage 2002;15:273-289.

7. Amidi A, Hosseini SMH, Leemans A, et al. Changes in Brain Structural Networks and Cognitive Functions in Testicular Cancer Patients Receiving Cisplatin-based Chemotherapy. J Natl Cancer Inst 2017;109.

8. Fischer FU, Wolf D, Scheurich A, Fellgiebel A. Association of structural global brain network properties with intelligence in normal aging. PLoS ONE 2014;9:e86258.

9. Lo CY, Wang PN, Chou KH, Wang J, He Y, Lin CP. Diffusion tensor tractography reveals abnormal topological organization in structural cortical networks in Alzheimer's disease. J Neurosci 2010;30:16876-16885.

10. Hosseini H. GAT: a graph-theoretical analysis toolbox for analyzing between-group differences in large-scale structural and functional brain networks. PLoS ONE 2012;7(7).

11. Hosseini SM, Mazaika P, Mauras N, et al. Altered Integration of Structural Covariance Networks in Young Children With Type 1 Diabetes. Hum Brain Mapp 2016;37:4034-4046.

12. Bruno JL, Hosseini SMH, Saggar M, Quintin EM, Raman MM, Reiss AL. Altered Brain Network Segregation in Fragile X Syndrome Revealed by Structural Connectomics. Cereb Cortex 2017;27:2249-2259.

13. Hosseini SMH, Kesler SR. Influence of Choice of Null Network on Small-World Parameters of Structural Correlation Networks. PLoS ONE 2013;8:e67354.

14. Latora V, Marchiori M. Efficient Behavior of Small-World Networks. Physical Review Letters 2001;87: 198701.

15. Rubinov M, Sporns O. Complex network measures of brain connectivity: Uses and interpretations. NeuroImage 2010;52:1059-1069.

16. Bassett DS, Bullmore ET. Small-World Brain Networks Revisited. Neuroscientist 2017;23:499-516.

17. Kesler SR, Watson CL, Blayney DW. Brain network alterations and vulnerability to simulated neurodegeneration in breast cancer. Neurobiol Aging 2015;36:2429-2442.

18. Bullmore E, Sporns O. Complex brain networks: graph theoretical analysis of structural and functional systems. Nat Rev Neurosci 2009;10:186198.

19. Zigmond AS, Snaith RP. The Hospital Anxiety and Depression Scale. Acta Psychiatr Scand 1983;67:361-70.

20. Webster K, Cella D, Yost K. The Functional Assessment of Chronic Illness Therapy (FACIT) Measurement System: properties, applications, and interpretation. Health Qual Life Outcomes 2003;1:79.

21. Cohen S, Kamarck T, Mermelstein R. A global measure of perceived stress. J Health Soc Behav 1983;24(4):385-96.

22. Buysse DJ, Reynolds CF, Monk TH, Berman SR, Kupfer DJ. The Pittsburgh Sleep Quality Index: a new instrument for psychiatric practice and research. Psychiatry Res 1989;28:193-213.

23. Benedict R, Schretlen D, Groninger L, Brandt J. Hopkins Verbal Learning TestRrevised: normative data and analysis of inter-form and test- retest reliability. Clin Neuropsychol 1998;12:43-55.

24. Reitan R. Validity of the Trail Making Test as an indicator of organic brain damage. Percept Mot Skills 1958;8:271-276.

25. Wechsler D. Wechsler Adult Intelligence Scale - Fourth Edition. 4ed; San Antonio: TX; 2008.

26. Wechsler D. Wechsler Memory Scale - Third edition manual. 3ed. San Antonio: TX: 1997.
